# Characteristics of chronic obstructive pulmonary disease in Spain from a gender perspective

**DOI:** 10.1186/1471-2466-9-2

**Published:** 2009-01-02

**Authors:** Pilar Carrasco-Garrido, Javier de Miguel-Díez, Javier Rejas-Gutierrez, Antonio Martín-Centeno, Elena Gobartt-Vázquez, Valentin Hernandez-Barrera, Angel Gil de Miguel, Rodrigo Jimenez-Garcia

**Affiliations:** 1 School of Health Sciences, Universidad Rey Juan Carlos, Alcorcón (Madrid), Spain; 2 Department of Pneumology, Hospital General Universitario Gregorio Marañón, Madrid, Spain; 3 Department of Health Outcomes Research, Medical Unit, Pfizer España, Alcobendas (Madrid), Spain; 4 Medical Unit, Pfizer España, Alcobendas (Madrid), Spain; 5 Medical Department, Boehringer Ingelheim España, San Cugat del Vallés (Barcelona), Spain

## Abstract

**Background:**

The objective of this study was to analyze the clinical and management characteristics of chronic obstructive pulmonary disease (COPD) in men and women, to determine possible gender-associated differences between the two groups of patients.

**Methods:**

An observational and descriptive epidemiological study (EPIDEPOC study). The study included patients with stable COPD and aged ≥ 40 years, evaluated in primary care. Data were collected relating to sociodemographic variables, clinical characteristics, quality of life (SF-12), severity of disease and treatment. The results obtained in men and women were compared.

**Results:**

A total of 10,711 patients (75.6% males and 24.4% females) were evaluated. Significant differences were found between males and females in relation to the following parameters: age (67.4 ± 9.2 years in men vs 66.1 ± 10.8 in women, p < 0.05), smoking (91.9% of the men were smokers or ex-smokers vs 30% of the women), comorbidity (the frequency of hypertension, diabetes, anxiety and depression was greater in women, while ischemic heart disease was more common in men), mental component of quality of life (49.4 ± 10.3 in men vs 44.6 ± 11.9 in women, p < 0.05) and severity of disease (56.5 ± 13.3% in men vs 60.7 ± 3.2 in women, p < 0.05). As regards treatment, the percentage use of long-acting b_2_-adrenergic agonists, anticholinergic agents, theophyllines and mucolytic agents was significant greater in men. The total annual cost of COPD was greater in males than in females (1989.20 ± 2364.47 € vs 1724.53 ± 2106.90, p < 0.05).

**Conclusion:**

The women with COPD evaluated in this study were younger, smoked less and have more comorbidity, a poorer quality of life, and lesser disease severity than men with COPD. However, they generated a lesser total annual cost of COPD than men.

## Background

In recent years there has been an increase in the incidence, prevalence and mortality of chronic obstructive pulmonary disease (COPD) in women [1,2]. Indeed, in some countries such as the United States, Canada, the United Kingdom or Finland, the absolute number of cases of the disease, as well as the number of hospital admissions and deaths, has been greater in women than in men [1,3-5]. This situation has developed despite the fact that classically COPD has been less probably diagnosed in women than in men [6,7].

Recent evidences have found that males and females might show a fenotipically different respond to tobacco smoke, showing men more propensities to develop an emphysema profile of the disease, while women could show mainly more airway affectation. However, as COPD is an inflammatory condition, a sexual dimorphism could be responsible for the different immunological response observed in the human being according to gender [8].

A recent study has shown that among patients with stable COPD, the women smoke less, are comparatively younger, and have less comorbidity than men with the same degree of airways obstruction [9]. However, the mentioned study has some limitations. Firstly, the patients were recruited exclusively from pneumology clinics, and thus possibly were not representative of the overall COPD population. Secondly, the female data could be applied only to those cases of smoking-related COPD, and not to those attributable to exposure to risk factors other than smoking. Third, since women have greater bronchial hyper-responsiveness than men [10], the exclusion of patients with symptoms of asthma or with a positive bronchodilator response may have introduced bias in the population.

The present study was made to evaluate the existence of gender differences in the clinical expression, diagnosis and management of COPD in a non-selected population of patients with the disease, recruited in the primary care setting.

## Methods

### Study design and population

This study forms part of the EPIDEPOC survey, an observational and descriptive, multicenter epidemiological study conducted in the primary care setting to explore the use of health care resources and assess the quality of life of patients with stable COPD [11].

Patient recruitment and calculation of the sample size corresponded to that conducted in the EPIDEPOC study. For calculation of sample size, a cluster design was used, considering 3 types of variables: health centers, physicians, and medical records. As the health centers were considered to be homogeneous and representatives of the Spanish geographical population, the medical record was chosen as the unit of study and the prescriber as the cluster. A previous study in a large cohort of 1,510 primary care patients found that the average annual cost per patient varied widely, with an estimated standard deviation of €3,407 [6]. Assuming a precision of €90, 5,505 medical records needed to be evaluated. If the effect of the cluster design is also taken into account, i.e., the loss of efficacy from the use of clusters, assuming a correlation of 0.3 and a cluster size of 5, a total of 2,422 prescribers and 12,111 medical records would be required. The subjects were consecutively included by primary care physicians in all the Spanish Autonomous Communities, with a distribution proportional to the population in each Community. The patients were recruited during a period of three months (from January 1 to March 31, 2003).

The study included patients of either sex or aged over 40 years, with a diagnosis of COPD established at least 12 months before the start of the study. The diagnosis of COPD was based on the criteria of the Spanish Society of Pneumology and Chest Surgery (*Sociedad Española de Neumología y Cirugía Torácica, SEPAR*) according to the forced spirometric demonstration of a forced expiratory volume in one second (FEV_1_) of less than 80% the corresponding reference value, and a FEV_1_/forced vital capacity (FVC) ratio of under 0.7 following bronchodilator testing. The severity of COPD was classified into three groups according to the FEV_1_: mild (FEV_1_: 60–80% of the reference value), moderate (FEV_1_: 40–59% of the reference value), and severe (FEV_1_: less than 40% of the reference value), based on the criteria of the SEPAR [12].

Patients with neurological or psychiatric disease not allowing adequate evaluation at the time of the study were excluded. Patients with COPD exacerbation in the previous month were likewise excluded. Exacerbation was defined as worsening of the clinical condition of the patient, in the form of increased expectoration, purulent sputum production, increased baseline dyspnea, or any combination of these symptoms.

The study was approved by the ethics committee of the *Fundación Hospital Alcorcón*, and verbal informed consent was obtained in all cases.

### Evaluation of the patients

A single visit was made, in all cases documenting the sociodemographic parameters (including gender), the year of diagnosis of COPD, the severity of the disease, and the use of health care resources in the previous 12 months. Associated comorbidity was also recorded, including the presence or absence of diabetes mellitus. The SF-12 quality of life questionnaire (an abridged 12-item version of the SF-36) was administered to all patients [13]. These 12 items explain more than 90% of the variance of the physical and mental components of the SF-36. They can be applied to calculate two scores: physical (PCS-12) and mental (MCS-12), using a value of 50 with a standard deviation (SD) of 10 as reference population. The SF-12 scores from 0 to 100, where the higher the score, the better the patient health condition.

The direct costs were calculated from the information relating to the different procedures, supplied by the management authorities of Area 8 Health Care (Madrid) and of the Alcorcón and Móstoles Hospitals. The "human capital" method was used to compute the indirect costs. This method uses as basic hypothesis the equivalence between the value of lost production and the wage associated with such production. In other words, one day off work implies a production loss equivalent to the wage corresponding to that same day. The data on employment and wages was obtained from the Spanish National Statistical Institute (*Instituto Nacional de Estadística, INE*) [14].

### Statistical particulars

The SPSS version 12.0 statistical package for Microsoft Windows was used throughout. Qualitative variables are given as frequencies and percentages, and quantitative variables as mean, standard deviation, minimum and maximum values. The Pearson χ^2 ^test was used to analyze the relationship between qualitative variables. Normality (Kolmogorov-Smirnov test), has been evaluated for all the quantitative variables. If the variables did fit the normality the Student t-test or ANOVA test for independent measures was used to calculate the differences between the means of two or more groups in the bi-variant analysis. If the normality could not be assumed nonparametric methods (Mann-Whitney *U *test or Kruskal-Wallism test) were used.

Finally in order to control the confounding effect of co-variables all comparisons between males and females have been conducted using multivariate models (logistic regression for binary variables and multiple lineal regression models for continuous variables). In all such cases age and severity of were included as possible confounders. Unfortunately the information about the physician who collected the 5 patients was not included in the database. Therefore we could not analyse the data using logistic regression with random effects. Statistical significance was accepted for p < 0.05.

## Results

A total of 10,711 patients (75.6% males and 24.4% females) were evaluated. The mean age was 67.1 ± 9.66 years, and was significantly greater in the male subgroup than among the women (67.40 ± 9.20 vs 66.13 ± 10.82, p < 0.05). Statistically significant differences were also seen between males and females as refers to smoking. In the male subgroup, 8.1% were non-smokers, 70.6% were ex-smokers, and 21.3% were active smokers. In the female subgroup, 70% were non-smokers, 17.7% were ex-smokers, and 12.3% were active smokers. Table 1 shows the sociodemographic characteristics of the study population according to gender and smoking habit.

**Table 1:** Sociodemographic characteristics of the studied COPD patients, according to gender and smoking habit.

VARIABLE	Male non-smokers	Male smokers	Female non-smokers	Female smokers
Age (mean ± SD) + † Ŧ	(647) 66.99 ± 10.06	(7356) 67.47 ± 9.13	(1803) 68.58 ± 10.08	(773) 60.21 ± 10.26
				
Occupational status:				
- Inactive (%)	(440) 73.9	(5184) 77.6	(1417) 84.7	(428) 58.9
- Active (%) + † Ŧ	(155) 26.0	(1494) 22.4	(256) 15.3	(298) 41.0
				
Educational level:				
- Patients without basic schooling (%)	(77) 14.7	(1142) 18.3	(536) 35.4	(85) 13.2
- Primary (%)	(308) 58.7	(3630) 58.1	(812) 53.7	(304) 47.1
- Secondary (%)	(103) 19.6	(1151) 18.4	(138) 9.1	(178) 27.6
- University (%) + † Ŧ	(37) 7.0	(326) 5.2	(27) 1.8	(79) 12.2

* Significant difference in the comparison of male smokers versus male non-smokers.

+ Significant difference in the comparison of female smokers versus female non-smokers.

† Significant difference in the comparison of male smokers versus female smokers.

Ŧ Significant difference in the comparison of male non-smokers versus female non-smokers.

The mean FEV_1 _was 57.4 ± 13.4%. The degree of airways obstruction was significantly greater in men than in women (56.64 ± 13.35% vs 60.72 ± 13.26%, p < 0.05). As regards comorbidity, the women showed a higher frequency of hypertension (49.5% vs 47.1% in men, p < 0.05), diabetes mellitus (18.4% vs 16.4%, p < 0.05), anxiety (36.7% vs 17.6%, p < 0.05), depression (23.1% vs 9.5%, p < 0.05) and allergy (13.2% vs 5.7%, p < 0.05). In contrast, males showed a comparatively higher frequency of heart disease (19.6% vs 16.3% in women, p < 0.05) and peptic ulcer (18.8% vs 12.7%, p < 0.05). As to health-related quality of life, after adjusting by age and severity, there were no significant gender differences in the scores corresponding to the physical component (35.96 ± 9.94 in men vs 35.91 ± 9.91 in women), though the gender differences were significant in the case of the mental component (49.41 ± 10.33 in men vs 44.66 ± 11.97 in women). Table 2 reports the health profile, the severity of airways obstruction, comorbidity, and quality of life of the studied COPD patients, according to gender and smoking.

**Table 2:** Health profile, severity of airways obstruction according to FEV_1_, co-morbidity, and quality of life (generic SF-12 questionnaire) of the studied COPD patients, according to gender and smoking habit.

Parameter	Male non-smokers	Male smokers	Female non-smokers	Female smokers
Physical exercise: * † Ŧ				
- None (%)	(153) 21.9	(2233) 30.6	(758) 42.5	(232) 30.4
- Light (%)	(436) 68.1	(4735) 64.8	(981) 55.0	(474) 62.0
- Moderate (%)	(51) 8.0	(334) 4.6	(43) 2.4	(58) 7.6
				
Obesity: + † Ŧ				
- Normal weight (%)	(130) 20.6	(1616) 22.3	(445) 25.6	(279) 36.6
- Overweight (%)	(382) 60.6	(4130) 57.0	(786) 45.2	(342) 44.8
- Obese (%)	(118) 18.7	(1489) 20.6	(509) 29.2	(142) 18.6
				
Severity of obstruction (according to FEV1) * + †				
Mild	(270) 44.1	(2276) 32.2	(695) 40.7	(342) 46.4
Moderate	(296) 48.4	(3906) 55.2	(861) 50.5	(353) 47.9
Severe	(46) 7.5	(896) 12.7	(150) 8.8	(42) 5.7
				
Comorbidity:				
- Hypertension (%) * + † Ŧ	(229) 40.0	(3259) 47.8	(899) 53.8	(277) 39.3
- Hypercholesterolemia (%) *	(197) 35.2	(2833) 42.3	(668) 41.0	(262) 38.0
- Heart disease (%) + †	(84) 15.4	(1294) 19.9	(301) 18.9	(65) 9.8
- Gastroduodenal ulcer (%) * †	(75) 13.7	(1254) 19.2	(207) 13.1	(78) 11.7
- Depression (%) † Ŧ	(46) 8.5	(619) 9.6	(353) 22.2	(168) 25.2
- Anxiety (%) † Ŧ	(89) 16.2	(1145) 17.7	(568) 35.6	(264) 39.4
- Diabetes(%) + Ŧ	(71) 13.0	(1089) 16.7	(322) 20.1	(96) 14.4
SF12 mental component (mean ± SD) † Ŧ	(647) 50.3 ± 9.8	(7356) 49.3 ± 10.4	(1803) 44.5 ± 12.0	(773) 44.9 ± 12.1
SF12 physical component (mean ± SD) * + † Ŧ	(647) 38.1 ± 10.0	(7356) 35.8 ± 9.9	(1803) 34.6 ± 9.6	(773) 38.9 ± 9.8

Normal weight (BMI < 27), Overweight (BMI ≥ 27 < 30), Obesity (BMI ≥ 30).

* Significant difference in the comparison of male smokers versus male non-smokers.

+ Significant difference in the comparison of female smokers versus female non-smokers.

† Significant difference in the comparison of male smokers versus female smokers, after adjusting by age and severity

Ŧ Significant difference in the comparison of male non-smokers versus female non-smokers, after adjusting by age and severity.

The men received a larger number of drugs for COPD than the women. Thus, men used an average of 2.32 ± 1.03 drugs, compared with 2.17 ± 1.04 in the case of the women – the difference being statistically significant. Likewise significantly greater among males was the percentage use of long-acting b_2_-adrenergic agonists (9.8% vs 7.9% in females, p < 0.05), anticholinergic drugs (85.6% vs 82.4%, p < 0.05), theophyllines (13.2% vs 7.6%, p < 0.05) and mucolytic agents (9.3% vs 7.7%, p < 0.05). However, no gender differences were recorded in the frequency of administration of corticoids – both inhalatory (22.1% in males vs 22.2% in females) and oral (4.4% vs 5.3%). As to vaccination antecedents, men received the antiinfluenza vaccine during the last campaign more often than women (88.1% vs 84.7%, p < 0.05). Table 3 reports the drug treatment and vaccinations of the study population according to gender and smoking habit.

**Table 3:** Drug treatment and vaccinations of the studied COPD patients, according to gender and smoking habit.

Treatment	Male non-smokers	Male smokers	Female non-smokers	Female smokers
Short-acting β2-adrenergic agonists (%)*	(216) 33.4	(2927) 39.8	(685) 38.0	(289) 37.4
Long-acting β2-adrenergic agonists (%)	(53) 8.2	(735) 10.0	(149) 8.3	(55) 7.1
Anticholinergic agents (%)†	(549) 84.8	(6304) 85.7	(1489) 82.6	(635) 82.1
Theophyllines (%)†	(64) 9.9	(986) 13.4	(155) 8.6	(42) 5.4
Inhalatory corticoids (%)+†Ŧ	(123) 19.0	(1648) 22.4	(441) 24.4	(130) 16.8
Oral corticoids (%)	(27) 4.2	(331) 4.5	(105) 5.8	(31) 4.0
Mucolytic agents (%)†	(50) 7.7	(692) 9.4	(151) 8.4	(48) 6.2
Antiinfluenza vaccine in last campaign (%)+†	(547) 85.3	(6409) 88.3	(1585) 88.9	(568) 74.8
Antipneumococcal vaccination at some time in past (%)+†	(210) 33.5	(2332) 32.8	(618) 35.5	(150) 20.2

* Significant difference in the comparison of male smokers versus male non-smokers.

+ Significant difference in the comparison of female smokers versus female non-smokers.

† Significant difference in the comparison of male smokers versus female smokers, after adjusting by age and severity

Ŧ Significant difference in the comparison of male non-smokers versus female non-smokers, after adjusting by age and severity.

Utilization of ressources in the last year was greater among male smokers than in male non-smokers. Differences were also detected in the number of primary care visits between male and female smokers (Table 4).

**Table 4:** Utilization of health care resources in the previous year among the COPD patients according to gender and smoking habit.

Health care resources	Male non-smokers	Male smokers	Female non-smokers	Female smokers
No. visits to primary care * † Ŧ	(625) 5.91 ± 5.67	(7045) 6.77 ± 5.75	(1724) 6.69 ± 5.72	(731) 6.18 ± 5.30
No. visits to pneumology clinic *	(602) 1.30 ± 1.53	(6838) 1.47 ± 1.54	(1640) 1.37 ± 1.40	(706) 1.32 ± 1.59
No. visits to emergency service * Ŧ	(581) 1.70 ± 0.81	(6608) 1.90 ± 0.87	(1592) 1.84 ± 0.86	(684) 1.84 ± 0.89
No. hospital admissions *	(523) 0.36 ± 0.83	(6026) 0.54 ± 1.27	(1429) 0.44 ± 0.93	(598) 0.41 ± 0.94
Duration hospital stay (days) *	(262) 5.74 ± 10.49	(3325) 7.67 ± 11.54	(773) 6.26 ± 10.37	(335) 6.27 ± 14.00

* Significant difference in the comparison of male smokers versus male non-smokers.

+ Significant difference in the comparison of female smokers versus female non-smokers.

† Significant difference in the comparison of male smokers versus female smokers, after adjusting by age and severity.

Ŧ Significant difference in the comparison of male non-smokers versus female non-smokers, after adjusting by age and severity.

The total annual cost of COPD management per patient was greater in males than in females (1989.20 ± 2364.47€ vs 1724.53 ± 2106.90 €, p < 0.05). Differences were likewise detected between male smokers and female smokers (2023.29 ± 2384.22 € vs 1696.15 ± 2165.94 €, p < 0.05). Table 5 shows the cost of the different health care resources utilized by the COPD patients according to gender and smoking habit.

**Table 5:** Cost of health care resources utilized among the COPD patients according to gender and smoking habit.

Cost component	Male non-smokers	Male smokers	Female non-smokers	Female smokers
Visits to primary care physician (€) * † Ŧ	(647) 95.77 ± 95.17	(7356) 108.81 ± 97.16	(1803) 107.25 ± 96.68	(773) 97.92 ± 89.58
Visits to pneumologist (€) †	(647) 85.35 ± 107.16	(7356) 96.44 ± 108.63	(1803) 88.01 ± 98.77	(773) 85.21 ± 110.45
Visits to emergency service (€) * Ŧ	(647) 101.45 ± 158.97	(7356) 139.67 ± 199.43	(1803) 125.73 ± 179.95	(773) 129.12 ± 209.41
Hospital admission (€) * †	(647) 584.04 ± 1532.18	(7356) 849.24 ± 1827.62	(1803) 685.60 ± 1598.17	(773) 642.26 ± 1702.83
Diagnostic tests (€) †	(647) 117.77 ± 147.87	(7356) 133.08 ± 149.89	(1803) 121.44 ± 136.29	(773) 117.58 ± 152.41
Respiratory drugs (€) †	(647) 471.97 ± 401.21	(7356) 502.24 ± 415.07	(1803) 477.62 ± 409.10	(773) 443.92 ± 387.19
Oxygen therapy (€) * †	(647) 54.72 ± 223.56	(7356) 105.88 ± 312.46	(1803) 77.00 ± 271.35	(773) 48,93 ± 219.68
Sick leave (€) + †	(647) 56.81 ± 280.13	(7356) 70.16 ± 291.70	(1803) 35.39 ± 205.88	(773) 118.63 ± 339.38
Antiinfluenza vaccination (€) + †	(647) 0.18 ± 0.55	(7356) 0.20 ± 0.58	(1803) 0.17 ± 0.54	(773) 0.37 ± 0.75
Antipneumococcal vaccination (€) + †	(647) 4.76 ± 6.87	(7356) 4.65 ± 6.83	(1803) 5.03 ± 6.96	(773) 2.84 ± 5.80
Total annual cost per COPD patient (€) * †	(647) 1585.93 ± 2085.04	(7356) 2023.29 ± 2384.22	(1803) 1736.78 ± 2078.85	(773) 1696.15 ± 2165.94

* Significant difference in the comparison of male smokers versus male non-smokers.

+ Significant difference in the comparison of female smokers versus female non-smokers.

† Significant difference in the comparison of male smokers versus female smokers, after adjusting by age and severity.

Ŧ Significant difference in the comparison of male non-smokers versus female non-smokers, after adjusting by age and severity.

## Discussion

The main finding of this study is that there are differences in the sociodemographic characteristics, associated diseases, quality of life, treatment, utilization of resources and cost of COPD according to patient gender and smoking habit. The true strength of the survey is found in the large number of patients involved, and in the fact that these were real-life subjects not included in a clinical trial but recruited in the primary care setting.

A number of previous studies have reported gender differences in the clinical presentation, diagnosis, treatment and prognosis of COPD [15-20]. Thus, it has been shown that women have more respiratory symptoms than men, with increased airways responsiveness, lower quality of life questionnaire scores, lesser response to prolonged treatment with exercise, and a more favorable prognosis at the time of starting oxygen therapy.

Of note in the present study is the large proportion of non-smoking women. No evaluation has been made of passive exposure to tobacco smoke, though women are known to be more exposed, and are more sensitive to exposure, than men [21]. In any case, earlier studies have shown the implication of other risk factors in the pathogenesis of COPD, including genetic factors, infections, environmental pollution, and occupational exposure [22]. Thus, as an example, a recent study has demonstrated a relationship between exposure to the smoke of burning wood or coal and the development of COPD [23]. The fact that our patients had been diagnosed with COPD for one year and presented spirometric evidence of airways obstruction upon inclusion in the study, increases the reliability of our findings. Another possibility is that a proportion of the non-smoking women in our series may actually not have COPD but other disorders characterized by airways obstruction, such as asthma. In fact, the percentage of an allergy history was higher in the female group. In this sense, no bronchodilator tests were carried out in this study, though it has been shown that unless full obstruction reversion is achieved, the mentioned test shows poor performance in discriminating between COPD and asthma [24]. In any case, the balance of error tends to tip in the opposite direction, i.e., it is more common to diagnose women with respiratory symptoms of asthma than of COPD [20,25], in the same way as among non-smokers [26]. On the other hand, and as has been commented above, in the evaluation of the clinical course, the subjects with asthma features were excluded; as a result, the impact of asthma upon the end results is scantly relevant.

Some studies suggest that women are more susceptible to the deleterious effects of smoking. A number of publications suggest that women have lung function similar to that of males at a younger age and with a lesser smoking history [9,27-32]. Gender-associated differences have also been found in the gradual worsening of lung function in smokers with COPD [33]. Thus, the annual decline in lung function in women has been associated with the degree of airways obstruction – an effect not seen in males.

Some authors report that women have fewer associated diseases than males with the same degree of airways obstruction [9]. In this study it has been shown that in general hypertension and diabetes mellitus is more common in women, while men show a larger proportion of other disorders such as hypercholesterolemia and ischemic heart disease – with differences in both cases according to smoking status. In addition, anxiety and depression were more frequent in women. Di Marco et al. also observed that women with COPD appear to be more exposed to psychological problems – these being related to symptomatic aspects of the disease such as dyspnea [34]. Lastly, and in coincidence with earlier studies reporting gender-associated differences in atopic markers [35-37], we recorded an increased allergy history in women compared with men. Other authors have published similar results [38,39].

As regards treatment, earlier studies have reported an increased frequency of inhalatory corticoid use among women, with a comparatively lower percentage use of theophyllines [40]. We likewise observed a lesser percentage use of theophyllines among women than in men – possibly in relation to the lesser severity of airways obstruction among the former. In relation to oxygen therapy, it has been shown that the mortality risk among COPD patients administered such therapy is greater in women than in men [41]. An explanation for such a poorer prognosis among women could be that the systemic complications of COPD, such as muscle dysfunction or depression, are more frequent in women and involve a poorer patient course [42].

Gender-associated differences were also observed in utilization of health resources. In fact, women have been shown to make more frequent utilization of health care services than men [43]. It also has been suggested that the risk for hospital admission is greater among women, and that the number of admissions due to COPD will gradually increase among females in the coming years [43,44]. In our study, gender differences were seen in the frequency of visits to the primary care physician – with more frequent visits among male smokers than in female smokers – and in the number of visits to the emergency service (greater in non-smoking women than in non-smoking men).

As refers to health-related quality of life, some authors have found that, for one same FEV1 level, women yield poorer scores in all domains of the St. George questionnaire [9]. In addition, the factors associated to quality of life have been shown to vary between sexes. Thus, in males, the main predictors include dyspnea, exercise capacity, the degree of hyper-insufflation, and comorbidity – while in women the main predictors are dyspnea and arterial oxygenation [45]. Other studies have reported poorer scores in the quality of life questionnaires in women compared with men [16-34,46-49]. In our study, women yielded lower scores in the mental component of quality of life, but not in the physical dimension. However, gender differences were obtained in this parameter according to patient smoker status. As regards the financial factors, we found the costs associated with COPD to be generally higher in males – particularly among the smokers. In contrast, other studies have reported no gender-associated differences in the costs associated with this disease [50]. These results should be taken into account when designing specific treatment strategies for different groups of COPD patients.

A possible limitation of our study is that, as commented previously in the method section, we did not use logistic regression with random effects. Ignoring clustering and unequal probability of selection of participants in our analyses may result in biased estimates [51,52].

## Conclusion

In conclusion, there are gender-related differences in the clinical characteristics, management, quality of life, and costs of COPD. The women with COPD evaluated in this study were younger, smoked less and have more comorbidity, a poorer quality of life and lesser disease severity than men with COPD. In addition, they used fewer drugs and health care resources than males – though there were also differences between the two gender groups according to smoker status.

## Competing interests

JRG and AMC are employees at Pfizer Spain and EGV is employee at Boehringer Ingelheim SA. The other authors have not any conflict of interest with Pfizer or Boehringer Ingelheim SA. This study has been funded by an unrestricted grant from Pfizer Spain and Boehringer Ingelheim SA.

## Authors' contributions

PCG, JRG, VHB, AGM and RJG have made substantive intellectual contributions to conception and design, acquisition of data and analysis and interpretation of data. PCG, JRG, JMD, AMC, AGM, and EGV have been envolved in drafting the manuscript and revising it critically for important intellectual content. All authors have given final approval of the version to be published.

## Pre-publication history

The pre-publication history for this paper can be accessed here:


